# Preliminary Insights into Quality of Life and Dietary Intake in Patients with Breast Cancer on Adjuvant Endocrine Therapy

**DOI:** 10.3390/cancers17132154

**Published:** 2025-06-26

**Authors:** Snjezana Petrovic, Danijela Ristic-Medic, Marija Paunovic, Biljana Pokimica, Milica Kojadinovic, Milan Gojgic, Aleksandra Arsic, Vesna Vucic

**Affiliations:** 1 Group for Nutritional Biochemistry and Dietology, Center of Research Excellence in Nutrition and Metabolism, Institute for Medical Research—National Institute of Republic of Serbia, University of Belgrade, Dr Subotica 4, 11000 Belgrade, Serbia; danijelar@imi.bg.ac.rs (D.R.-M.); marija.paunovic@imi.bg.ac.rs (M.P.); biljana.pokimica@imi.bg.ac.rs (B.P.); milica.kojadinovic@imi.bg.ac.rs (M.K.); aleksandra.arsic@imi.bg.ac.rs (A.A.); vesna.vucic@imi.bg.ac.rs (V.V.); 2 Department of Surgical Oncology, University Hospital Medical Center “Bezanijska Kosa”, Zorza Matea bb, 11070 Belgrade, Serbia; gojgic.milan@bkosa.edu.rs

**Keywords:** breast cancer, endocrine therapy, aromatase inhibitors, tamoxifen, quality of life, dietary intake

## Abstract

**Background:** Endocrine therapies are accompanied by side effects that significantly impact the quality of life (QoL) of women with breast cancer. Adequate diet is important for fulfilling nutritional requirements, preserving health, and supporting therapy in this vulnerable population. **Methods**: This preliminary study evaluated the QoL of life and dietary intake in 185 women with breast cancer on two therapies, aromatase inhibitors (AIs) and tamoxifen, using the Functional Assessment of Cancer Therapy—Endocrine Symptoms (FACT-ES), European Organization for Research and Treatment of Cancer Quality of Life Questionnaire (EORTC QLQ-C30), and Breast Cancer Specific Questionnaire (QLQ-BR23) and a 24 h dietary recall. A total of 185 women were included in the study and fulfilled the FACT-ES, of whom 73 fulfilled other two questionnaires and a 24 h recall. **Results**: No significant differences were found in the overall QoL between groups. Joint pain (95.3%) and reduced libido (84.7%) were most common with AIs, while tamoxifen users more frequently reported weight gain and irritability (93.0%, each), and vasomotor and gynecological symptoms. Macronutrient intake was similar, though AIs users consumed more energy-dense (*p* ≤ 0.001) and sugary foods (*p* = 0.034), while tamoxifen users had higher omega-6 PUFA intake. Both groups exhibited suboptimal intake of vitamin D, calcium, and selenium, and a higher phosphorus consumption relative to recommended daily values. **Conclusions**: Preliminary findings showed that QoL and dietary intake were comparable between patients with BC on AIs and tamoxifen treatment. Endocrine-related symptoms were more prevalent among tamoxifen users, whereas joint pain was most common in AIs users. Nutritional interventions may be warranted in both groups to ensure adequate intake of essential micronutrients in accordance with recommended dietary guidelines.

## Simple Summary

This study looked at women with breast cancer who were taking hormone treatments, either aromatase inhibitors (AIs) or tamoxifen. These treatments can cause side effects that affect daily life. The researchers found that overall quality of life was similar in both groups, but the types of side effects were different. Women on AIs often had joint pain and low sex drive, while those on tamoxifen more often gained weight, felt irritable, and had hot flashes or other hormone-related symptoms. When it came to diet, both groups ate similarly, but AIs users tended to eat more sugary and high-calorie foods. Tamoxifen users ate more omega-6 fats. Many women in both groups were not obtaining enough important nutrients like vitamin D, calcium, and selenium. In short, while the treatments affected women differently, both groups could benefit from better nutrition to feel their best during therapy.

## 1. Introduction

Breast cancer (BC) is the second most prevalent malignancy globally and the leading cancer type among women. Annually, more than 2.3 million new cases are diagnosed, with 670,000 related deaths worldwide in 2022 (WHO) [1]. Patients diagnosed with BC face numerous challenges arising from both the tumor and the side effects of treatment. These challenges encompass a range of physical symptoms, including pain and fatigue, as well as emotional and psychological distress, such as diminished body image and impaired daily role functioning [2]. In addition to halting disease progression and enhancing survival rates, therapeutic interventions are designed to alleviate these symptoms and improve the overall quality of life (QoL) for BC patients.

The most prevalent subtype of BC is hormone-receptor-positive, including estrogen receptor-positive (ER+) and/or progesterone receptor-positive (PR+) tumors, which account for approximately 70% of all BC cases [3]. These cancers generally have a favorable prognosis and are treated with adjuvant hormone therapies, typically tamoxifen and aromatase inhibitors (AIs). Although hormone therapies are effective in prolonging both overall and recurrence-free survival, reducing recurrence rates, and inhibiting tumor growth [4,5], they are frequently associated with significant side effects. Common adverse effects of both tamoxifen and AIs include hot flashes, as well as joint and muscle pain. Tamoxifen is also linked to an increased risk of thromboembolism and endometriosis, while AIs therapy is associated with osteoporosis, myalgias, sleep disturbances, and sexual dysfunction [6,7]. These side effects substantially compromise the QoL of BC patients and are often the primary reasons for discontinuation of therapy [8].

Evidence suggests that appropriate nutritional interventions may alleviate many of the adverse effects associated with hormone-related therapies in BC patients [9,10]. In line with general dietary guidelines, adherence to a healthy dietary pattern, characterized by a high intake of fruits, vegetables, whole grains, poultry, and fish, alongside limited consumption of red meats, refined carbohydrates, sweets, and high-fat dairy products, has been associated with improved prognosis and overall survival in individuals diagnosed with BC [11,12]. Moreover, poor dietary quality following a BC diagnosis has been linked to increased risk of BC-specific mortality [13]. Despite growing evidence on the role of diet in patient outcomes [14], standardized nutritional guidelines specifically tailored for those undergoing hormonal therapy remain limited or insufficiently defined.

The aim of this study was to evaluate the quality of life of BC patients undergoing adjuvant endocrine therapy, as an integral component of long-term disease management, by comparing women receiving AIs and those on tamoxifen. As a pilot investigation, it also sought to explore dietary intake patterns through a pilot study using three 24 h dietary recalls, providing preliminary insights for future, larger-scale research.

## 2. Materials and Methods

### 2.1. Patients

The BC survivors on AIs and tamoxifen therapy were recruited between December 2023 and April 2025, from University Hospital Medical Center Bezanijska kosa, Belgrade, and Institute for Oncology and Radiology Serbia, Belgrade. The eligibility criteria were: histologically confirmed BC, stage I to IIIa; ER+/PR+, HER2-; women 45–70 years; 6–30 months on tamoxifen or AIs. Exclusion criteria encompassed the presence of other malignancies, serious chronic diseases, metastatic disease, or active infections. Our study enrolled only women with HER2-negative (HER2-) breast cancer to eliminate side effects from trastuzumab, a treatment for HER2-positive (HER2+) tumors. Trastuzumab has similar adverse effects to endocrine therapies, including fatigue, nausea, musculoskeletal pain, and cardiotoxicity [15], all of which can additionally impact the assessment of quality of life in women undergoing endocrine therapy. Accordingly, to maintain the validity of our findings, we excluded women undergoing trastuzumab therapy in combination with adjuvant treatment using AIs or tamoxifen. Participants were contacted by their medical oncologist and invited to complete the online Functional Assessment of Cancer Therapy—Endocrine Symptoms (FACT-ES) survey and to visit the Institute for Medical Research (IMR) at the University of Belgrade for a nutritional assessment. Each prospective participant received detailed information about the study along with the invitation. Informed consent was obtained electronically through the survey interface before participation. A total of 200 patients were contacted, of whom 185 completed the online survey—100 patients were undergoing tamoxifen therapy and 85 were receiving aromatase inhibitors (AIs). Among these, 73 patients visited the IMR in person, where they completed the European Organization for Research and Treatment of Cancer (EORTC) Quality of Life Questionnaire (QLQ-C30) and Breast Cancer Specific Quality of Life Questionnaire (QLQ-BR23), as well as a 24 h dietary intake assessment. Patients with disease progression or relapse were excluded at the clinical evaluation stage, as a change in therapy would have been required in such cases. This approach helped maintain the homogeneity of our study population in terms of treatment status, there by strengthening the reliability of our quality of life assessments. The study was approved by the Medical Ethics Committee of University Hospital Medical Center Bezanijska kosa, Faculty of Medicine, University of Belgrade (6041/1), Ethical committee of Institute for Medical Research, University of Belgrade (EO606/23), and Ethical committee of Institute for Oncology and Radiology (O1-1/2024/1443).

### 2.2. Quality of Life and Side-Effect Analysis

Quality of life was evaluated using three questionnaires: FACT-ES, QLQ-C30, and QLQ-BR23, all in the Serbian language.

The FACT-ES is specifically designed for women on hormonal therapy of BC [16]. This is a reliable instrument of in total 46 questions developed to assess physical, social, emotional, and functional well-being and measure endocrine-related side-effects including fatigue, vasomotor symptoms, vaginal symptoms, nervousness, and nausea in studies on patients with BC undergoing endocrine therapy. The results of FACT-ES are scored in a range of 0–200 for extended endocrine subscale (ESS-23, 23 items). The higher the score, the better the QoL. In our study, the scoring results were compared between the AIs and tamoxifen group to detect if any differences in QoL and drug-related side-effects among the treatments. The prevalences of symptoms were expressed as number and percentage of affected patients. The FACT-ES questionnaire was validated in Serbian between January and May 2024, initiated by the researchers involved in this study and conducted under the supervision of the Functional Assessment of Chronic Illness Therapy (FACIT) organization. The process included standard forward and backward translation and pilot testing on 10 BC patients to ensure linguistic and cultural accuracy. FACIT approved the Serbian version as a valid and reliable tool for assessing health-related quality of life in Serbian-speaking BC patients.

The QLQ-C30 and QLQ-BR23 were applied to further extend overall health and QoL measurements. Both questionnaires have been previously validated in Serbian (Crombach’s α = 0.844) and utilized in BC research [17,18]. QLQ-C30 is a validated questionnaire for assessing health-related QoL in cancer patients, used in a wide range of clinical trials and non-trial studies. It is composed of 30 items covering physical, role, emotional, cognitive and social functioning, a global health status and QoL, symptoms like fatigue, pain, nausea, and six single-item measures including dyspnea, appetite loss, sleep disturbance, constipation, diarrhea, and financial impact. QLQ-BR23 is a supplementary module employed in conjunction with the QLQ-C30 to provide detailed information relevant to evaluating QoL in patients with BC. It incorporates 23 items which are scored within five multi-item scales to assess side effects of systemic therapy, arm symptoms, breast symptoms, body image, and sexual functioning. In addition, single items measure future perspectives, sexual enjoyment, and hair loss. All measures range in score 0–100. For both instruments, QLQ-C30 and QLQ-BR23, a high score for a functional scale and global health status represents high QoL, while a high score for symptom items represents high level of problems. Furthermore, given the close connection between nutrition and overall health, nutritional intake in participants who completed QLQ-C30 and QLQ-BR23 was assessed using 24 h dietary recalls.

### 2.3. Dietary Intake

Dietary intake evaluation was based on participants’ subjective retrospective report, using the repeated 24 h recalls (two weekdays and one weekend day) as the assessment method. Data was collected within in-depth multiple-pass direct structured interviews, administered by skilled survey staff. The survey methods provided a better understanding of the patients’ eating habits during the week. Starting with the first item after waking up, patients recalled all food and beverage consumed during 24 h, and estimated portion sizes, such as number of pieces, slices, cups, spoons, etc. Food quantities were estimated using common household measures, kitchen tools, and packaging information. Daily intake of macro and micronutrients, as well as total energy intake, was quantified using Slovenian food composition database, complemented with customized recipes and specific brand information [19].

### 2.4. Sample Size

The sample size calculation was performed based on a study by Sert et al., who evaluated the effect of hormonal treatment on quality of life (QoL) in breast cancer patients [20]. We calculated that a minimum sample size of 42 subjects in each group would allow for the detection of differences in endocrine symptoms between AIs and tamoxifen users, with 80% power and a 5% significance level.

### 2.5. Statistical Analysis

Statistical analyses were conducted using SPSS version 23.0 (SPSS Inc., Chicago, IL, USA), with a significance level set at *p* ≤ 0.05. Data are expressed as numbers (percentages), mean ± standard deviation (SD) or median (IQR). The Kolmogorov–Smirnov test was used to assess data distribution. To compare the differences between the two groups, Student’s t test was used for normally distributed data and the Mann–Whitney U test was applied when data did not meet normality assumptions. For comparisons of categorical data, the Chi-square test was applied.

## 3. Results

### 3.1. Patients’ and Disease Characteristics

The study participants’ and disease characteristics are summarized in Table 1.

Among patients who completed the FACT-ES questionnaire (*n* = 85 in the AIs group and *n* = 100 in the tamoxifen group), the mean age was 57.83 ± 7.95 years for those receiving AIs and 51.48 ± 5.73 years for those on tamoxifen. This difference was statistically significant, indicating that patients treated with tamoxifen were significantly younger than those receiving AIs. A similar trend was observed among the subset of patients who completed all three QLQ questionnaires (*n* = 52 for the AIs group and *n* = 21 for the tamoxifen group), with mean ages of 58.98 ± 7.74 and 53.27 ± 6.71 years, respectively. However, almost all patients in both treatment groups were postmenopausal, the only exception was 9 out of 100 patients on tamoxifen therapy who completed FACT-ES questionnaire.

Regarding tumor stage, 22.3% of patients in the AIs group and 28% in the tamoxifen group were diagnosed with clinical stage IB disease. About half of the patients in both groups (56.4% in AIs group and 51% in tamoxifen group) had grade II tumors, while grade IIIA tumors were present in 21.2% of patients treated with AIs and in 21% of those receiving tamoxifen. All study participants were estrogen receptor (ER)-positive and human epidermal growth factor receptor 2 (HER2)-negative.

### 3.2. FACT-ES

In total, 185 women fulfilled FACT-ES questionnaire. Among them 100 were receiving tamoxifen and 85 AIs. FACT-ES subscales scores were assessed and compared between AIs and tamoxifen treated patients (Table 2).

With respect to ranges, all well-being scores for BC patients on endocrine therapy were in the upper-third of range for both treatments, with 18–21 points for PWB, SWB, and FWB out of a maximum of 28 points, and 16–17 points for EWB (maximum 24 points). Also, the FACT-ES 23 total score for both treatments was above 135 points out of 200. In general, the total scores and the scores for four FACT-ES subscale domains did not differ significantly between patients on AIs and tamoxifen therapy.

Table 3 displays the occurrences of adverse events in patients with BC on AIs and tamoxifen endocrine therapy, also assessed by FACT-ES, as part of additional concerns. The most common symptoms in the AIs group were joint pains (95.3%) and a loss of interest in sex (84.7%), followed by a bloated feeling and feeling irritable (76.3% each), while the patients on tamoxifen reported gain weight and feeling irritable (93.0% each), mood swings (90.0%), a bloated feeling, and loss of interest in sex (88.0% each), as a most frequent adverse events. Of the 19 symptoms shown, 9 symptoms were experienced by a larger proportion of patients in the tamoxifen group than in the AIs group. However, joint pains were significantly more frequently associated with AIs than with tamoxifen therapy (95.3% vs. 77%, *p* = 0.001).

### 3.3. Quality of Life QLQ-C30 and QLQ-BR23

The QoL questionnaires, EORTC QLQ-C30 and EORTC QLQ-BR23, were completed by 52 patients on AIs and 21 patients on tamoxifen. Similarly to FACT-ES well-being domains, the patients with BC on AIs and tamoxifen therapy did not differ in global health status assessed through QLQ-C30. Moreover, there was no difference in the presence of specific symptoms such as fatigue, dyspnea, insomnia, and constipation, as well as in body image and future perspective. However, breast symptoms were markedly worse in the tamoxifen group compared to the AIs group. Scores of selected EORTC QLQ-C30 and EORTC QLQ-BR23 items are presented in Table 4.

### 3.4. Dietary Intake

Dietary intake of patients with BC in AIs and tamoxifen group was assessed by three 24 h dietary recalls and presented in Table 5 and Table 6.

Energy intake, macronutrient distribution (carbohydrates, proteins, fats), and the intake of most micronutrients did not significantly differ between the two therapy groups. The median daily energy intake in AIs group was 1566 kcal, compared to 1539 kcal in the tamoxifen group. Similarly, intakes of carbohydrate, total dietary fiber, and types of protein (plant based vs. animal based) showed no notable differences.

However, daily dietary intake analysis showed several differences between the AIs and tamoxifen users (Table 5). Participants in the AIs group consumed foods with higher energy density (0.91 kcal/g vs. 0.52 kcal/g) and reported a great intake of sugar and sugar-rich foods (1.97 servings vs. 1.11 servings). In contrast, women in the tamoxifen group had a significantly higher intake of omega-6 PUFA averaging 10.89 g/day compared, 7.72 g/day in the AIs group. No significant differences were observed in the intake of total fat, saturated fatty acids (SFA), monounsaturated fatty acids (MUFA), or total PUFA.

Regarding micronutrient intake, no significant differences were observed between the groups for the majority of vitamins and minerals. However, the patients on AIs therapy had a significantly lower intake of α-carotene (198 µg vs. 336 µg), fluoride (163 µg vs. 270 µg), and biotin (24.7 µg vs. 35.4 µg) compared to those on tamoxifen. Intake of vitamins A, D, E, K, C, and the B-complex group (including B1, B2, niacin, B6, B12, and folic acid), as well as key minerals such as calcium, magnesium, iron, zinc, copper, sodium, potassium, selenium, and phosphorus did not differ significantly between the groups (Table 6).

When compared to established dietary reference values, such as European Food Safety Authority (EFSA) guidance [16], the intake of most micronutrients among both therapy groups was generally below recommended levels. While the intake of vitamin D was critically low across all patients (~2.9 µg), far below the adequate intake of 15 µg, the vitamin B6 intake in both therapy groups was slightly lower than the recommended 1.6 mg, and particularly in the AIs group (1.18 mg). α-carotene and fluoride intakes were also significantly lower in AIs users. Although α-carotene has no established dietary reference intake, the difference may reflect lower intake of carotenoid-rich foods. Fluoride intake in both groups was below the adequate intake of 3.5 mg, particularly in the patients on AIs therapy (163 µg vs. 270 µg; *p* ≤ 0.05). Calcium intake was markedly below the PRI of 950 mg in both groups (~600 mg), while the intake of zinc was at the lower end of the reference range (Table 6).

## 4. Discussion

Long-term endocrine therapy, often administered for five years or more, leads to sustained estrogen suppression, contributing to a broad spectrum of menopausal symptoms and a decline in QoL among patients with BC. Numerous studies have consistently reported lower QoL and a great symptom burden in patients with BC compared to healthy people [22,23]. Considering the well-established link between higher QoL and better adherence to endocrine therapy [24], regular assessment of QoL is crucial. Such evaluations not only provide insight into patients’ overall well-being but also play a key role in addressing symptom burden and preventing premature treatment discontinuation.

In this study, the focus was to evaluate the QoL of participants in the AIs and tamoxifen group. No significant differences were observed in physical (PWB), social (SWB), emotional (EWB), or functional well-being (FWB) domains. Across all dimensions, scores remained in the upper-third of the scale (>66.7%), suggesting a relatively good QoL among participants [25]. AIs-treated patients reported the highest scores in SWB (~21/28), while tamoxifen users scored highest in PWB (~20/28). Previous studies have similarly reported no differences in SWB, EWB, and FWB across therapies. However, reduced PWB among AIs users has been frequently linked to AIs-induced arthralgia [26]. In line with these findings, joint pain was more prevalent in our cohort among women receiving AIs therapy (95%) compared to those on tamoxifen (77%), supporting existing evidence of an increased musculoskeletal burden associated with AIs [27]. Notably, no significance was observed in the severity of arm symptoms between the groups. Interestingly, women in the AIs group reported significantly less severe breast-related symptoms and experienced these symptoms less frequently (37.6% vs. 57%, *p* = 0.013). These findings diverge from those of a recent French cohort study, which reported no differences in breast symptomatology between therapy groups [28].

Previous studies have highlighted the high prevalence of vasomotor symptoms associated with endocrine therapy [29,30], although some evidence suggests that AIs may be better tolerated than tamoxifen [31]. In our cohort, global health status, fatigue, and dyspnea scores did not differ significantly between therapy groups. However, vasomotor symptoms such as hot flashes, cold, and night sweats were reported less frequently among AIs users (72.8%, 51.8%, and 56.5%, respectively) compared to those receiving tamoxifen (94%, 80%, and 92%, *p* < 0.001).

Gynecological symptoms, including reduced libido (≥85%) and vaginal dryness (75%) were highly prevalent in both groups. These findings are consistent with previous research, which has identified vaginal dryness and dyspareunia as common side effects associated with AIs therapy. However, some studies suggest that these symptoms may be less pronounced in patients receiving tamoxifen [32]. Herein, vaginal discharge was more common in tamoxifen users (*p* < 0.001), while vaginal irritation showed a borderline significant difference between groups (*p* = 0.058).

Neurological symptoms, particularly mood swings and irritability, were significantly more prevalent in the tamoxifen group (90% and 93%, respectively) compared to AIs users (58.2% and 76.5%, *p* < 0.001 and *p* = 0.003). No significant differences were observed in the incidence of headaches, dizziness, insomnia, or in future perspectives. These findings may be attributed to estrogen’s role in mood regulation, as both treatments modalities are linked to affective disturbances [33,34]. A deeper understanding of the neuropsychological effects of endocrine therapies is crucial for improving patient management, optimizing therapeutic adherence and providing comprehensive support to individuals undergoing treatment.

Both AIs and tamoxifen treatments are associated with changes in body composition and weight [35]. In our study, weight gain was more prevalent in the tamoxifen group (83% vs. 69.4%, *p* < 0.001). Importantly, no notable differences were observed between the groups in the prevalence of other gastrointestinal symptoms such as bloating, diarrhea, or constipation. Weight changes during endocrine therapy are multifactorial, involving metabolic, behavioral, and psychological factors [36]. Nutritional interventions, including dietary supplementation (e.g., EPA and DHA), may alleviate treatment-related side effects and improve overall outcomes in patients with BC [37]. Furthermore, diets rich in plant-based foods and low in red meat and saturated fats have been linked to improved survival rates following BC diagnosis, underscoring the potential role of dietary modifications in enhancing treatment outcomes [38].

Daily dietary intake was generally consistent across both treatment groups. As previously stated, AIs substantially reduce body estrogen levels, a change that has been associated in the literature with mood disturbances, including anxiety and depressive symptoms [39]. These hormonal changes may contribute to increased cravings for sugar and energy-dense foods [40]. Regarding lipid intake, no significant differences were observed between the groups in terms of total fats, SFA, MUFA, and total PUFA. The only notable difference was a somewhat higher intake of omega-6 PUFA in the tamoxifen group. Although hormonal factors may influence food preferences, overall, the patterns observed were relatively consistent between treatment groups.

Nutritional quality of the diet plays an important role in controlling BC. A recent study presented significant association observed between BC and the index of nutrition quality of vitamin A, vitamin E, vitamin B6, riboflavin, vitamin K, biotin, vitamin B12, vitamin C, zinc, calcium, and magnesium [41]. Significantly lower dietary intake of α-carotene, fluoride, and biotin were observed in AIs users compared to tamoxifen users in our study. Although these nutrients are not directly linked to BC progression, α-carotene has antioxidant properties that could play a role in oxidative stress reduction, and biotin is involved in lipid metabolism and gene regulation [42,43]. These functions are supported by previous evidence, but their clinical relevance in the context of BC remains unclear. The lower intake of these micronutrients in the AIs group might reflect differences in food choices; however, this remains a hypothesis and could be influenced by treatment-related symptoms such as changes in taste or appetite. Endocrine therapy for BC, can lead to alopecia and changes in hair and skin texture due to hormonal alternations [44]. While biotin supplementation has been suggested to support hair growth and reduce hair thinning, current evidence on its role in BC patients remains limited and inconclusive [44,45]. Regarding bone health, the role of fluoride is still debated. Some studies suggested that fluoride may stimulate bone formation, potentially offering benefits for patients with reduced bone mineral density due to endocrine therapy [46]. Nevertheless, concerns about fluoride toxicity at elevated levels highlight the importance of cautious consideration in its use among women undergoing endocrine therapy for BC.

Many foods rich in omega-6 fatty acid, such as seeds, nuts, and legumes, are also notable sources of biotin [47], which may partly explain the higher intake of this vitamin observed in tamoxifen group. While previous studies have reported that elevated levels of omega-6 fatty acids may contribute to BC recurrence, this association remains inconclusive; the optimal balance between omega-3 and omega-6 fatty acid in women undergoing endocrine therapy is still not fully understood [48]. A balanced intake of both omega-6 and omega-3 fatty acids is generally recommended, as it has been associated with reduced inflammation and improved immune function, which may positively influence overall health during treatment [49,50]. However, direct evidence linking specific fatty acid ratios to improved outcomes in endocrine-treated BC patients remains limited.

Evidence supports micronutrient potential in alleviating common side effects, such as fatigue, bone loss, mood disturbances, and cognitive impairment, which are frequently reported during long-term hormonal treatment [51]. Although more research is needed to establish causality, integrating dietary strategies to optimize the intake of these essential nutrients may support patients’ well-being and promote better adherence to endocrine therapy for BC.

It is important to emphasize that both treatment groups in our study reported a suboptimal intake of a wide range of vitamins and minerals when compared to DRV nutrition recommendation [21].

Notably, the mean daily intake of vitamin D was approximately five times lower than the recommended 15 µg, while vitamin A intake reached only about 400 µg, compared to the target value of 650 µg. Similarly, folate and vitamin B12 intakes achieved only around 70% of the recommended daily intake levels. In addition, calcium intake was significantly below recommended values (approximately 600 mg vs. 950 mg) and selenium intake was similarly reduced (50 µg compared to the recommended 70 µg). In contrast, phosphorus intake exceeded the recommended 550 mg, nearly twofold in both groups.

A study conducted in women over the age of 40 reported a 32.5% prevalence of osteoporosis, which increased markedly with advancing age, reaching 68% in women older than 80 years [52]. In contrast, in our study, 67% of women had osteopenia or osteoporosis a proportion markedly higher than that reported by Vujasinovic et al. (40.91%) in a general population of women of the similar age [53]. Lower vitamin D and calcium status have been consistently associated with concurrent loss of muscle and bone mass in older adults [54], highlighting the established importance of these nutrients in this vulnerable population. However, the intake of both micronutrients in our study was far below the recommended values.

Particular attention has been given to vitamin D for its potential role in BC recurrence prevention. Several observational studies have shown that adequate serum concentrations of 25-hydroxyvitamin D [25 (OH)D] (>30 ng/mL) are associated with a lower risk of recurrence and improved overall survival in BC survivors [55]. Although these associations are well-documented, they do not establish causality. Vitamin D supplementation showed promise in some studies, with potential immunomodulatory and antioxidant effects, especially when used as part of combined interventions [51]. However, while some evidence suggests a possible benefit of vitamin D, the effects on tumor-related outcomes remain inconclusive. For example, a pilot randomized controlled trial by Tirgar et al. involving 88 BC patients has examined combined effects of vitamin D and synbiotics on inflammation and treatment response [56]. The intervention led to increased serum vitamin D levels, and improvements in anti-inflammatory markers such as IL-10, but no significant differences were observed in tumor growth markers, residual tumors, or metastasis. These findings point to a potential, but not yet fully understood, role of vitamin D in supporting overall health and immune balance during BC treatment. Further high-quality interventional studies are needed to clarify its clinical impact on cancer outcomes.

Endocrine therapy for BC, including AIs and tamoxifen, has been associated with alterations in immune function due to estrogen suppression [57], which may contribute to an increased susceptibility to infections, particularly respiratory and urinary tract infections [58]. Chronic excessive phosphorus intake may contribute to an increased risk of cardiovascular disease and systemic inflammation [59]. In our study, phosphorus intake exceeded the recommended levels in both groups. Maintaining an adequate intake of immune-relevant micronutrients such as vitamin D, zinc, and selenium is essential for optimal immune function [57,58,59,60,61]. Although direct evidence in BC survivors receiving endocrine therapy is limited, nutritional adequacy may play a supportive role in mitigating infection risk in these patients. Given the potential vulnerability of BC survivors undergoing endocrine therapy, maintaining adequate micronutrient status may support immune function and reduce infection risk. Routine nutritional assessment and individualized dietary guidance should be considered as part of comprehensive care in this patient group. Health authorities, such as the World Health Organization (WHO) and the American Institute for Cancer Research (AICR), recommend meeting nutritional needs primary through a balanced diet rather than supplements [62].

The principal limitation of this study is the reliance on self-reported QoL questionnaires, which are subjective and may be influenced by individual perception, mood, or social desirability bias. Furthermore, these tools may have limited sensitivity to subtle changes and may not fully capture all relevant aspects of QoL. Also, the limitation of this study is the potential recall bias inherent in the 24 h dietary recall method. Participants may have misreported their intake, often overestimating the consumption of nutrient-dense foods (e.g., fruits and vegetables) and underestimating the intake of energy-dense, nutrient-poor foods (e.g., sweets, snacks, and high-fat products), which may compromise the validity of the dietary data, although these data were collected in the presence of a qualified nutritionist. Another potential limitation of this study is the significant age difference between the treatment groups. However, this disparity is unlikely to have substantially influenced outcomes related to menopausal status, as all patients receiving AIs and the vast majority of those on tamoxifen were postmenopausal. The lack of adjustment for potential confounding variables, such as body mass index (BMI), physical activity levels, and comorbidities could also be a potential limitation of this preliminary study, and these factors should be accounted for in future analyses. An additional limitation is the relatively small sample size. However, a key strength of this study is the multicenter design involving patients from different geographic locations in Serbia, which increases the relevance of the findings to the national population.

## 5. Conclusions

Our study found no significant differences between AIs and tamoxifen in their impact on physical, social, emotional, and functional well-being or global health status among women with BC. However, differences emerged in the frequency and severity of specific symptoms, including joint pain, weight gain, hot flushes, cold and night sweats, mood swings, irritability, vaginal discharge, and breast-related symptoms, with most being more prevalent among tamoxifen users. Although minor differences in food choices were observed, macronutrient intake was comparable across treatment groups. AIs users consumed more energy-dense and sugar-rich foods, while tamoxifen users had a higher intake of omega-6 PUFA. Nevertheless, both groups failed to meet the recommended daily intake for several key micronutrients, including vitamin D, calcium, selenium, and phosphorus. Future studies involving larger cohorts are needed to further explore QoL and dietary intake in women undergoing endocrine therapy, to refine comparisons between AIs and tamoxifen, and to validate our preliminary observations. These insights may help healthcare professionals tailor dietary treatment strategies to better preserve QoL in patients receiving endocrine therapy for breast cancer.

## Figures and Tables

**Table 1:** Baseline characteristics.

		Aromatase Inhibitors (AIs)		Tamoxifen	
		*n* = 85	*n* = 52	*n* = 100	*n* = 21
Age, Mean ± SD		57.83 ± 7.95	58.98 ± 7.74	51.48 ± 5.73 ***	53.27 ± 6.71 *
Menopausal status, No. (%)	Pre-	0	0	9 (9)	0
	Post-	85 (100)	52 (100)	91 (91)	21 (100)
Overall stage (clinical), No. (%)	IB	19 (22.3)	14 (26.9)	28 (28)	6 (28.6)
	IIA	20 (23.5)	10 (19.2)	24 (24)	3 (14.3)
	IIB	28 (32.9)	16 (30.1)	27 (27)	7 (33.3)
	IIIA	18 (21.2)	12 (23.1)	21 (21)	5 (23.8)
Breast markers	ER/PR Positive HER2 Negative	85 (100) 85 (100)	52 (100) 52 (100)	100 (100) 100 (100)	21 (100) 21 (100)

ER—Estrogen receptor; PR—Progesterone receptor; HER2—Human epidermal growth factor receptor 2. Statistical significance * *p* ≤ 0.05, *** *p* ≤ 0.001, compared to AIs (*n* = 85) and AIs (*n* = 52), respectively.

**Table 2:** Well-being scores and total FACT-ES score in patients with BC on AIs and tamoxifen therapy.

	Aromatase Inhibitors (AIs) Mean ± SD (*n* = 85)	Tamoxifen Mean ± SD (*n* = 100)	*p* Values
PWB subscale score ^a^	19.61 ± 5.05	20.91 ± 5.16	0.138
SWB subscale score ^a^	20.17 ± 5.12	19.64 ± 5.33	0.367
EWB subscale score ^b^	16.79 ± 4.71	17.13 ± 4.08	0.745
FWB subscale score ^a^	18.79 ± 4.89	18.95 ± 4.86	0.973
ESS-23 ^c^	62.48 ± 14.47	59.25 ± 13.50	0.083
FACT-ES 23 total score ^d^	137.87 ± 28.18	135.88 ± 26.78	0.466

FACT-ES—Functional Assessment of Cancer Therapy-Endocrine Symptoms; PWB—Physical Well-Being; SWB—Social/Family Well-Being; EWB—Emotional Well-Being; FWB—Functional Well-Being; ESS—Endocrine Symptom Subscale; FACT-ES 23 total score—PWB + SWB + EWB + FWB + ESS-23; Results are presented as means ± SD. ^a^ Score 0–28; ^b^ Score 0–24; ^c^ Score 0–92; ^d^ core 0–200. Statistical significance *p* ≤ 0.05, calculated either by Student’s *t*-test or Mann–Whitney U test, depending on data distribution.

**Table 3:** Endocrine symptoms in patients with BC on AIs and tamoxifen therapy.

Symptoms	Aromatase Inhibitors (AIs) No. (%) (*n* = 85)	Tamoxifen No. (%) (*n* = 100)	*p* Values
Vasomotor symptoms			
Hot flushes (ES1)	62 (72.9)	94 (94)	<0.001
Cold sweats (ES2)	44 (51.8)	80 (80)	<0.001
Night sweats (ES3)	48 (56.5)	92 (92)	<0.001
Neuropsychological symptoms			
Lightheaded (An9)	46 (54.1)	56 (56)	0.914
Headaches (An10)	49 (57.6)	68 (68)	0.193
Mood swings (ES12)	58 (58.2)	90 (90)	<0.001
Feeling irritable (ES13)	65 (76.5)	93 (93)	0.003
Gastro-intestinal symptoms			
Gained weight (ES10)	59 (69.4)	93 (83)	<0.001
Vomiting (O2)	12 (14.1)	6 (6)	0.108
Diarrhea (C5)	24 (28.2)	21 (21)	0.332
Bloated Feeling (Tax1)	65 (76.5)	88 (88)	0.061
Gynecologic symptoms			
Vaginal discharge (ES4)	35 (41.2)	70 (70)	<0.001
Vaginal irritation (ES5)	29 (34.1)	49 (49)	0.058
Vaginal bleeding (ES6)	4 (4.7)	10 (10)	0.281
Vaginal dryness (ES7)	54 (75.3)	75 (75)	0.126
Discomfort intercourse (ES8)	56 (65.9)	65 (65)	1.000
Lost interest in sex (ES9)	72 (84.7)	88 (88)	0.662
Breast tenderness (ES11)	32 (37.6)	57 (57)	0.013
Joint pains			
Joint pains (BRM1)	81 (95.3)	77 (77)	0.001

Statistical significance (*p* ≤ 0.05) calculated by chi-square test.

**Table 4:** EORTC QLQ-C30 and EORTC QLQ-BR23 scores in patients with BC on AIs and tamoxifen therapy.

	Aromatase Inhibitors (AIs) Median (IQR) or Mean ± SD (*n* = 52)	Tamoxifen Median (IQR) or Mean ± SD (*n* = 21)	*p* Values
EORTC QLQ-C30			
Global health status ^a^	61.38 ± 16.11	67.46 ± 17.66	0.179
Fatigue ^b^	44.99 ± 24.84	40.28 ± 33.43	0.563
Dyspnea ^b^	16.67 (41.67)	0.00 (16.67)	0.958
Insomnia ^b^	66.67 (33.33)	33.33 (50.00)	0.455
Constipation ^b^	0.00 (33.33)	16.67 (33.33)	0.155
EORTC QLQ-BR23			
Arm Symptoms ^b^	33.33 (33.33)	21.11 (19.44)	0.152
Breast Symptoms ^b^	8.33 (25.00)	29.17 (45.84)	0.001
Body Image ^a^	83.33 (41.67)	79.16 (52.08)	0.631
Future perspective ^a^	66.67 (33.33)	33.33 (66.67)	0.054

EORTC QLQ-C30—European Organization for Research and Treatment of Cancer Quality of Life Questionnaire; EORTC QLQ-BR23—European Organization for Research and Treatment of Cancer Breast Cancer Specific Quality of Life Questionnaire. Results are presented as means ± SD or median (IQR). ^a^ Score 0–100, a higher score indicates a better level of functioning and quality of life; ^b^ Score 0–100, a higher value indicates more severe symptoms. Statistical significance *p* ≤ 0.05, calculated either by Student’s *t*-test or Mann–Whitney U test, depending on data distribution.

**Table 5:** Dietary intake of macronutrients in patients with BC undergoing AIs or tamoxifen therapy.

Daily Dietary Intake	Aromatase Inhibitors (AIs) Median (IQR) or Mean *±* SD (*n* = 52)	Tamoxifen Median (IQR) or Mean *±* SD (*n* = 21)	*p* Values
Energy intake (kcal)	1566 (404)	1539 (409)	0.474
Energy density (kcal/g)	0.91 (0.58)	0.52 (0.22)	0.001
Carbohydrate (g)	176 ± 48.0	167 ± 46	0.315
Total fiber (g)	21.8 ± 7.88	20.6 ± 5.28	0.782
Sugar and sugar rich foods (serving)	1.97 (3.07)	1.11 (1.54)	0.031
Protein (g)	66.2 (22.26)	61.1 (17.9)	0.900
Protein animal (g)	38.6 ± 25.5	40.9 ± 11.6	0.615
Protein plant (g)	25.0 ± 8.38	24.0 ± 8.41	0.615
Fats (g)	65.8 (23.8)	66.1 (21.9)	0.890
SFA (g)	16.8 (9.9)	19.7 (4.5)	0.280
MUFA (g)	21.9 (10.2)	21.0 (14.9)	0.890
PUFA (g)	12.1 (7.54)	15.0 (5.99)	0.395
omega-6 PUFA (g)	7.72 (6.44)	10.9 (4.37)	0.034
omega-3 PUFA (g)	1.42 (1.34)	1.76 (2.21)	0.177
Cholesterol (mg)	261 (176)	312 (84.5)	0.108

IQR—interquartile range. SFA—Saturated fatty acids; MUFA—monounsaturated fatty acids; PUFA—polyunsaturated fatty acids. Statistical significance *p* ≤ 0.05.

**Table 6:** Dietary intake of micronutrients in patients with BC undergoing AIs or tamoxifen therapy.

Daily Dietary Intake	Aromatase Inhibitors (AIs) Median (IQR) or Mean *±* SD (*n* = 52)	Tamoxifen Median (IQR) or Mean *±* SD (*n* = 21)	*p* Values	DRV- EFSA Guidance [21]
Vitamin A, µg	400 (373)	432 (249)	0.379	**650** µg
α-carotene, µg	198 (532)	336 (592)	0.050	N/A
β-carotene, µg	2078 (1848)	2220 (1962)	0.436	N/A
Vitamin D µg	2.95 (2.77)	2.91 (4.59)	0.372	15 µg
Vitamin E, mg	11.4 (7.74)	12.6 (5.00)	0.597	11 mg
Vitamin K, µg	92.2 (117)	108 (48.0)	0.223	70 µg ^a^
Vitamin C, mg	110 (88.0)	95.5 (60.6)	0.597	95 mg
Vitamin B1, mg	0.99 ± 0.52	1.04 ± 0.25	0.985	**0.1** mg ^b^
Vitamin B2, mg	1.26 (0.65)	1.34 (0.34)	0.821	**1.6** mg
Niacin (B3), mg	14.7 (7.55)	15.3 (5.70)	0.563	**1.6** mg ^c^ NE/MJ
Vitamin B6, mg	1.18 (0.59)	1.42 (0.54)	0.481	1.6 mg
Biotin, µg	24.7 (19.9)	35.4 (12.1)	0.059	40 µg
Folic acid, µg	240 (86.7)	232 (95.3)	0.589	**330** µg DFE
Vitamin B12, µg	2.97 (0.38)	2.25 (1.72)	0.905	4 µg
Calcium, mg	603 (289)	605 (253)	0.649	950 mg
Phosphorus, mg	1055 (466)	978 (349)	0.959	550 mg
Magnesium, mg	274 (116)	287 (128)	0.687	300 mg
Iron, mg	12.4 ± 3.62	12.8 ± 3.22	0.755	11 mg ^†^
Zinc, mg	7.20 ± 2.50	7.70 ± 1.72	0.301	9.3–12.7 mg ^d^
Copper, mg	1365 (529)	1318 (645)	0.594	1300 mg
Sodium, mg	1604 (933)	1788 (773)	0.405	2000 mg
Potassium, mg	2346 (804)	2352 (889)	0.979	3500 mg
Selenium, µg	51.2 (45.6)	47.8 (32.8)	0.886	70 µg
Fluoride, µg	163 (136)	270 (201)	0.050	3.5 mg ^e^

EFSA—European Food Safety Authority; DRV—dietary reference value; IQR—interquartile range; DFE—Dietary Folate Equivalents; NE—Niacin Equivalents; N/A—Not available information. Population Reference Intakes (PRIs) are presented in bold type and Adequate Intake (AI) in ordinary type. ^a^ Recommended to take 1 µg/kg of body weight, assuming an average person weighs 70 kg; ^b^ it is calculated based on caloric intake, at 0.4 mg per 1000 kcal, an average intake of 2000 kcal was taken; ^c^ it is calculated based on caloric intake, at 6.6 mg per 1000 kcal, an average intake of 2000 kcal was taken; ^d^ 7.5 mg recommendation for women with moderate folate intake, 12.7 mg for women with high folate intake; ^e^ recommended to take 0.05 µg/kg of body weight, assuming an average person weighs 70 kg; ^†^ postmenopausal woman recommendation. Statistical significance *p* ≤ 0.05.

## Data Availability

The data that support the findings of this study are available from the corresponding author upon reasonable request.
